# The influence of lattice misfit on screw and edge dislocation-controlled solid solution strengthening in Mo-Ti alloys

**DOI:** 10.1038/s43246-023-00353-8

**Published:** 2023-04-17

**Authors:** Georg Winkens, Alexander Kauffmann, Johannes Herrmann, Andreas K. Czerny, Susanne Obert, Sascha Seils, Torben Boll, Carolina Baruffi, You Rao, William A. Curtin, Ruth Schwaiger, Martin Heilmaier

**Affiliations:** 1 Institute for Applied Materials (IAM), Karlsruhe Institute of Technology (KIT), Engelbert-Arnold-Straße 4, 76131 Karlsruhe, Germany; 2 Laboratory for Multiscale Mechanics Modeling, École Polytechnique Fédérale de Lausanne, 1015 Lausanne, Switzerland; 3 Institute of Energy and Climate Research (IEK), Forschungszentrum Jülich GmbH, 52425 Jülich, Germany; 4 Chair of Energy Engineering Materials, RWTH Aachen University, 52056 Aachen, Germany

**Keywords:** Metals and alloys, Theory and computation

## Abstract

Mo-Ti alloys form solid solutions over a wide range of compositions, with lattice misfit parameters increasing significantly with titanium content. This indicates a strong increase in the critical stress for edge dislocation motion. Here, we probe the transition from screw to edge dislocation-dominated strengthening in Mo-Ti solid solutions with titanium content up to 80 at%. The alloys were scale-bridging characterized to isolate the impact of substitutional solid solution strengthening. Mechanical testing yielded no significant influence of grain boundaries or grain orientation. The results were corrected for the strengthening by unavoidable interstitial oxygen. Modelling of screw and edge dislocation-controlled solid solution strengthening was applied to the results to evaluate the contributions of both dislocation types. The analysis reveals that screw dislocation motion controls the strength in allows with less than 40 at% titanium, while edge dislocation motion provides comparable strength for 60–80 at% titanium. These results in a system of reduced chemical complexity support the recent investigations of edge dislocation-controlled strengthening found in high-entropy alloys.

## Abstract (web summary)

In body-centered cubic alloys, screw dislocations are considered to be strength-controlling. Here, a systematic investigation of Mo-Ti alloys with varying lattice misfit reveals a transition from screw to edge dislocation-controlled strength.

## Introduction

In body-centered cubic (BCC) metals, screw dislocations are considered to be deformation rate limiting due to their higher critical stress for motion compared to edge dislocations^1^. Solutes lead to an efficient increase of this critical stress for dislocation glide by solid solution strengthening. In most models for BCC alloys, solid solution strengthening is only discussed based on the motion of screw dislocations (see Refs. ^2–4^ for example). However, the development of medium and high entropy alloys (HEA,^5–7^) within the last decades as examples for complex concentrated solid solution yielded new interest in solid solution strengthening. In particular, theoretical and experimental evidence on the relevance of edge dislocations on solid solution strengthening in BCC HEAs was found^8–15^.

One possible explanation for the relevance of edge dislocations is a large lattice misfit of the constituent solute atoms, which increases the critical stress significantly stronger for edge compared to screw dislocations^16,17^. Following these considerations, a transition between screw and edge dislocation-based strengthening might be observed when the lattice misfit surpasses a certain threshold as recently proposed^18^. However, a systematic investigation of the correlation of strengthening contributions by edge and screw dislocations to the lattice misfit has not been reported yet.

With an increasing number of constituent elements, the possibility for interactions among them increases, which might lead to superimposed effects from e.g., short-range ordering^19^ and precipitation^20^.

**Fig. 1:**
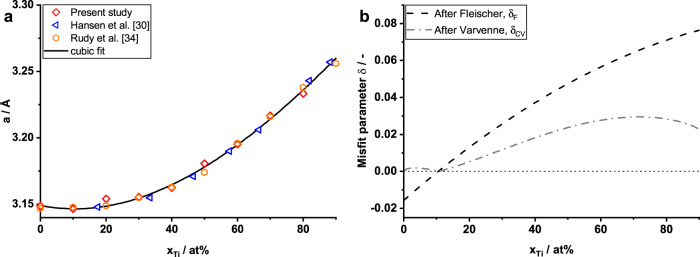
Lattice parameter and misfit in Mo-Ti solid solutions. **a** Lattice parameter *a* of Mo-Ti solid solutions as a function of the Ti concentration *x*_Ti_. Present study (red symbols) in comparison to results by Hansen et al. (blue)^30^ and Rudy et al. (orange)^34^ and a cubic fit (black line). **b** The lattice misfit parameters for the Mo-Ti system based on the cubic fit from **a** after Fleischer^16, 22^ (black dashed line) and after Varvenne^17^ (gray dash-dotted line). The dotted line indicates zero misfit.

Thus, fundamental relations are better investigated in a system of reduced chemical complexity compared to HEAs. The Mo-Ti system meets significant requirements for such an objective: Mo-Ti solid solutions can be synthesized in a single-phase BCC structure over a large range of compositions under practical cooling conditions^21^. Due to the strong non-linearity of the lattice parameter (see Fig. 1a), the lattice misfit changes strongly as a function of solute concentration. This allows for the investigation of strengthening in the cases of small and large misfits within a single alloy system. Figure 1b shows the values for two different misfit parameters. The lattice misfit parameter defined by Fleischer^22^, ${\delta }_{{{{{{{{\rm{F}}}}}}}}}=\frac{1}{a} \, \frac{{{{{{\partial}}}}} \, a}{{{{{{\partial}}}}} \, {x}_{{{{{{{{\rm{Ti}}}}}}}}}}$ often serves as a reference value. It describes the relative expansion of the lattice parameter *a* as a function of solute atom concentration *x*_i_ and is used in the models by Fleischer^22^ and Labusch^16^ in the dilute limit. An early attempt to model solid solution strengthening in HEAs also adopted this parameter^23^. The misfit parameter defined by Varvenne et al.^17^, ${\delta }_{{{{{{{{\rm{CV}}}}}}}}}=\frac{1}{3\bar{V}}\sqrt{{\sum }_{n}{x}_{n}\,\Delta {V}_{n}^{2}}$ properly quantifies the volumetric misfit of each atom in a solid solution compared to a virtual “average matrix”, and so is appropriate across the entire concentration range. This misfit parameter is the basis for a recently developed model for solid solution strengthening in all HEA, and so can also be applied to concentrated binary solid solutions^18^.

In the present study, Mo-Ti solid solutions were synthesized in the range of 0–80 at% Ti and scale-bridging characterized to reveal the exclusive contribution of substitutional solid solution strengthening. Mechanical tests were performed on several length scales to determine superimposed effects of grain boundaries and grain orientation. To evaluate the strengthening contributions of screw and edge dislocations, the recent model-compatible descriptions of solid solution strengthening by Maresca and Curtin^12,24,25^ were applied to the results. Since Ti is susceptible to O intake^26^, which increases the strength of its alloys significantly^27,28^, chemical and microstructural investigations were carried out to assess the interstitial O content. The impact of O content on the strength of the Mo-Ti solid solutions was evaluated and corrected for using a Labusch-like model. At Ti concentrations ≤40 at%, screw dislocation motion controls the strength of Mo-Ti solid solutions, while edge dislocation motion provides comparable strength for 60–80 at%.

## Results and discussion

### Crystal structure

The X-ray diffraction (XRD) patterns reveal a BCC structure for all alloys (Strukturbericht A2, W prototype, space group no. 229) in line with an earlier report on arc-melted Mo-Ti alloys^21^. The equilibrium phase diagram shows a miscibility gap and a eutectoid decomposition in the range of studied Mo-Ti solid solutions^29^. Necessary annealing durations to achieve equilibrium were for example 650 h at 600 °C^30^ or 120 h at 750 °C^31^, while the samples presented here were subjected to fast cooling rates, both in the water-cooled Cu crucible after arc melting as well as after the homogenization treatments during furnace cooling. Thus, there are no indications for either of the two reactions. Martensitic transformation and the formation of *ω* phase were not observed in the studied solid solutions, consistent with reported concentration limits (*x*_Mo,mart_ < 6 at%^32^ and *x*_Mo,*ω*_ < 15 at%^33^).

Atom probe tomography (APT) data was analyzed to determine indications of phase separation. The Pearson correlation coefficients indicate a random distribution of atoms in both alloys, see Table 1. A nearest-neighbor analysis in Mo-80Ti revealed a slightly smaller average distance between identical atoms as compared to randomized data, see Table 1. While this might be a first indication of beginning ordering, the shift is not sufficient to perform a meaningful cluster analysis.

**Table 1:** Pearson correlation coefficients (PCC) for the element distribution in both alloys investigated in APT.

	Mo-10Ti		Mo-80Ti	
	Mo	Ti	Mo	Ti
PCC/–	0.02	0.03	0.15	0.04
${\bar{d}}_{{{{{{{{\rm{ss}}}}}}}}}$/pm	–	–	658 ± 3	207 ± 1
${\bar{d}}_{{{{{{{{\rm{rs}}}}}}}}}$/pm	–	–	673 ± 2	210 ± 1

For Mo-80Ti, NNA was performed to determine the average distance between ions of the same species ${\bar{d}}_{{{{{{{{\rm{ss}}}}}}}}}$ in comparison to randomized data ${\bar{d}}_{{{{{{{{\rm{rs}}}}}}}}}$.

### Lattice parameters

The lattice parameters determined from the patterns match the results published in refs. ^30,34^, see Fig. 1a. Recent data published by Kim et al.^35^ is uniformly higher than the results shown here and is not considered further. Investigations on the lattice parameter of Ti-9Mo as a function of O concentration yielded only a marginal increase for 1.6 at% O^28^, similar to the statistical uncertainty of the present analysis. The impact of O on the lattice parameter is therefore neglected. The behavior is strongly non-linear, having nearly zero slope at low Ti concentrations and then increasing sharply to a near-linear behavior above 50 at%. This system thus shows a very notable deviation from a linear behavior. Figure 1a also shows a cubic fit to all data as

1 $$a=\left(-0.0572\,{x}_{{{{{{{{\rm{Ti}}}}}}}}}^{3}+0.2430\,{x}_{{{{{{{{\rm{Ti}}}}}}}}}^{2}-0.0493\,{x}_{{{{{{{{\rm{Ti}}}}}}}}}+3.1492\right)\mathring{\rm A}$$

The misfit quantity suggested by Fleischer, ${\delta }_{{{{{{{{\rm{F}}}}}}}}}=\frac{1}{a}\,\frac{\partial \,a}{\partial \,{x}_{{{{{{{{\rm{n}}}}}}}}}}$,^22^ is shown in Fig. 1b for historical purposes; it is not useful for any model predictions. The solid solution strengthening model for edge dislocation-controlled strengthening includes the misfit quantity ${\delta }_{{{{{{{{\rm{CV}}}}}}}}}=\frac{1}{3\bar{V}}\sqrt{{\sum }_{n}{x}_{n}\Delta {V}_{n}^{2}}$. In the binary Mo-Ti alloys, this parameter is simplified to ${\delta }_{{{{{{{{\rm{CV}}}}}}}}}=\frac{1}{a}\,\frac{\partial \,a}{\partial \,{x}_{{{{{{{{\rm{Ti}}}}}}}}}}\,\sqrt{{x}_{{{{{{{{\rm{Ti}}}}}}}}}\,(1-{x}_{{{{{{{{\rm{Ti}}}}}}}}})}$, which is also shown in Fig. 1b. Both misfit quantities in Fig. 1b include derivatives of the lattice parameter as a function of solute concentration. Hence, the misfit values are very sensitive to the precise fit to the data in the region 40–50 at% Ti, where the derivative changes from low to high values. This will impact the predictions of edge dislocation strengthening in this region. Using the present polynomial fit, *δ*_CV_ reaches a value of nearly 0.03 in the range 60–80 at% Ti.

### Young’s modulus

**Fig. 2:**
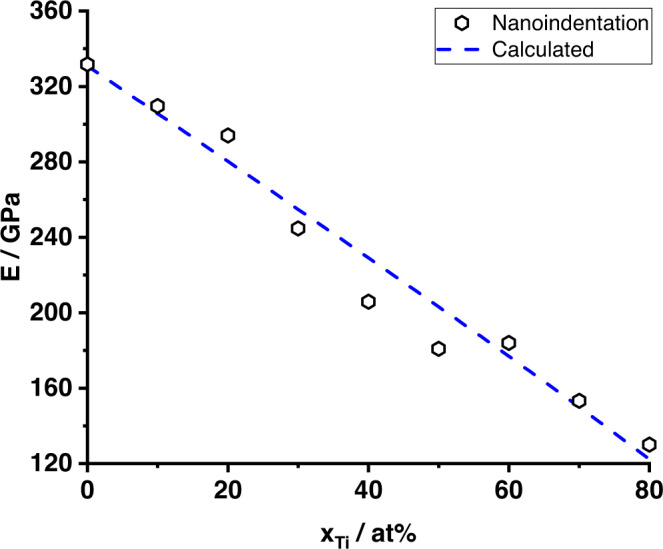
Experimental and calculated Young’s modulus. The Young’s modulus measured by nanoindentation (black symbols) and the calculation (blue dashed line) using weighted single crystal constants of Mo^37^ and Ti^38^. The standard deviation was smaller than the icon size and therefore omitted.

The Young’s modulus determined from nanoindentation decreases almost linearly from 332 GPa for Mo to 130 GPa for Mo-80Ti, see Fig. 2. The standard deviation of each measurement was smaller than its icon size and is therefore omitted in the figure. Additionally, Young’s modulus $\bar{E}$ was calculated as $\bar{E}=2\,\bar{G}(1+\bar{\nu })$ from the shear modulus $\bar{G}$, bulk modulus $\bar{B}$ and Poisson’s ratio $\bar{\nu }$ based on single crystal stiffnesses, since these quantities are also included in the edge dislocation strengthening model^36^:

2 $$\bar{G}=\sqrt{\frac{1}{2}{\bar{C}}_{44}({\bar{C}}_{11}-{\bar{C}}_{12})};\quad \bar{B}=({\bar{C}}_{11}+2{\bar{C}}_{12})/3;\quad \bar{\nu }=\frac{3\bar{B}-2\bar{G}}{2(3\bar{B}+\bar{G})}$$

For the calculations, concentration-weighted single crystal stiffnesses for Mo (*C*_11_ = 463 GPa, *C*_12_ = 161 GPa, *C*_44_ = 109 GPa^37^) and Ti (*C*_11_ = 134 GPa, *C*_12_ = 110 GPa, *C*_44_ = 36 GPa^38^) were used. The results are also presented in Fig. 2 and agree well with the experimental data. The strongest deviations are obtained in the concentrated solid solutions with ≈ 20 GPa, which might indicate a deviation of the simple linear rule of mixture for concentrated solutions.

Kim et al.^35^ recently reported data on the shear moduli of Mo-Ti alloys. When the obtained data on Young’s modulus and the shear modulus data published by Kim et al. are used to estimate Poisson’s ratio of the Mo-Ti alloys, values close to *ν* = 0.5 are obtained. This is unreasonably high for BCC metals and alloys. For comparison, Poisson’s ratio using the results from Eq. (2) yields plausible values of *ν*_Mo_ = 0.3, *ν*_Mo−80Ti_ = 0.36 and *ν*_Ti_ = 0.41, respectively. Therefore, the data for the shear moduli presented in ref. ^35^ are inconsistent with the present Young’s modulus data and are not considered further.

### Mechanical testing

**Fig. 3:**
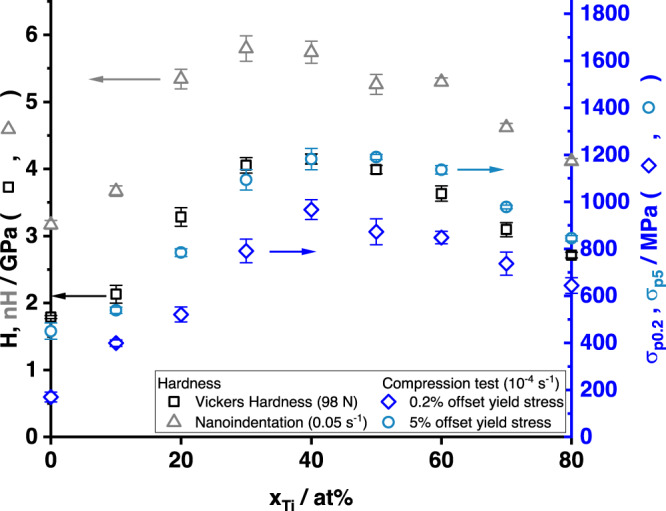
Mechanical properties of Mo-Ti solid solutions. Vickers hardness *H* and nanohardness *nH* (black squares and gray triangles, respectively, left axis) and 0.2% and 5% offset yield strength *σ*_p0.2_ and *σ*_p5_ (dark blue diamonds and light blue circles, respectively, right axis) as a function of Ti content *x*_Ti_ for the Mo-Ti alloys.

The results of the nanoindentation (*nH*) and Vickers hardness testing (*H*), as well as the *σ*_p0.2_ and *σ*_p5_ from macroscopic compression testing are shown in Fig. 3. Nanohardness and Vickers hardness data share the scale on the left in Fig. 3. They can be related as *nH* = 1.07 *H* + 1.36 GPa (${R}_{{{{{{{{\rm{adj}}}}}}}}}^{2}=0.93$, see Fig. 7). All data sets show an approximately linear increase of strength or hardness with increasing Ti concentration for Mo-rich solid solutions. For Ti-rich solid solutions, the strength or hardness also increases with increasing solute content, but with a smaller slope. The peak strength or hardness is detected at 30 or 40 at% Ti, which will be rationalized later. There is no softening observed. The results on Vickers hardness are consistent with published data^35^. Nanoindentation hardness measurements yield results independent from grain boundary effects, but only from a few grains. Vickers hardness results are obtained from many grains of various orientations, but can be increased by oxide particles at grain boundaries. Although data for Mo suggests only a small impact by grain boundary strengthening^39^, no data for Mo-Ti alloys is available. As the grain and indent sizes are similar, an influence by grain boundary strengthening also needs to be considered. The offset between both data sets is attributed to the indentation size effect, where the hardness detected in indentation experiments depends on the indentation depth^40,41^. The good correlation indicates that neither oxide particles, grain size nor grain orientation have a significant impact on the hardness testing results from the present study.

Since hardness testing involves considerable plastic straining, hardness cannot be directly recalculated into initial yield stress. Hardness usually correlates to the flow stress at approximately 8% plastic strain^42^. In the present study, 5% plastic strain was consistently achieved in all samples and, thus, the 5% offset yield strength *σ*_p5_ is shown in Fig. 3 using the scale on the right. The scale on the right has been chosen so that *H* and *σ*_p5_ are matched across the entire concentration range, and the resulting correlation is *σ*_p5_ = 0.285 *H*, (${R}_{{{{{{{{\rm{adj}}}}}}}}}^{2}=0.84$, see Fig. 7). This is very close to the suggested value of 0.3^42^.

The models predict the yield strength of alloys. As the stress-strain curves are continuous without pronounced yield phenomenon, the 0.2% offset yield strength *σ*_p0.2_ is presented in Fig. 3 using the right axis. This quantity is compared to the models in sections “Screw dislocation strengthening” and “Edge dislocation strengthening.” Yield strength and *σ*_p0.2_ are typically several hundred MPa lower than *σ*_p5_ as expected for alloys that show moderate work hardening rates. An apparent relationship between *H* and *σ*_p5_ can be obtained, and such a relationship is often used to estimate *σ*_p0.2_ but this is generally inaccurate since the relationship depends on the work hardening (and hence the difference between *σ*_p5_ and *σ*_p0.2_).

### Interstitial atom contents

**Fig. 4:**
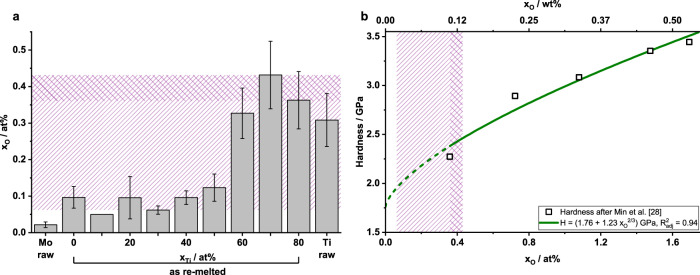
Interstitial O and its correction. **a** The O contents of the alloys determined by HCGE in at%. N contents were below 200 at-ppm for all alloys. **b** Vickers hardness *H* of quenched Ti-9Mo solid solutions as a function of O content *x*_O_, taken from ref. ^28^. The green line shows a Labusch-like fit to the data. The dashed part indicates the extrapolation to zero O. As reference, the top axis shows the O content *w*_O_ in wt%. The range of O correction by extrapolation is shown as single-hatched area in both images. The double-hatched area indicates the range of O correction by interpolation.

Hot carrier gas extraction (HCGE) yielded increasing O contents in the samples from about 0.1 at% in low-Ti alloys (corresponding to 180 wt-ppm in Mo-10Ti) to an O content of at least 0.3 at% in the high-Ti-containing samples (corresponding to 770 wt-ppm in Mo-70Ti), see Fig. 4a. O contents in the Mo-10Ti sample were below the detection limit, therefore this detection limit is presented to indicate the maximum amount of O that may be present in the sample. For comparison, results for Mo and Ti before arc-melting are also shown in Fig. 4a. N content was between 100 and 200 at-ppm (corresponding to 10 to 40 wt-ppm) for all alloys. The impact of such low N content is not considered in the further analysis.

In order to reveal the amount of dissolved O compared to O bonded in Ti oxides, APT analysis was performed on samples of Mo-10Ti and Mo-80Ti. In the APT measurements of Mo-10Ti, no significant amount of O is detected. In contrast, the entire O is dissolved in the Mo-80Ti solid solution (0.33 at% determined by APT and (0.36 ± 0.08) at% by HCGE. Errors in APT measurements were smaller than the indicated decimal places and are omitted). For both samples, no significant N content was detected in APT. These results are compatible with results by Bryant^43^, who investigated the amount of dissolved oxygen in refractory metal solid solutions based on the valence electron per atom ratio $\frac{e}{a}$. For solid solutions with $\frac{e}{a}\ge 5.7$, no dissolved O was experimentally detected. The threshold of 5.7 is reached at 15 at% Ti, if 6 and 4 valence electrons are assumed for Mo and Ti, respectively. In agreement with these results, no interstitial O was detected in Mo-10Ti ($\frac{e}{a}=5.8$) using APT, while the entire O was dissolved in Mo-80Ti ($\frac{e}{a}=4.4$). Therefore, only the offset yield strength values for alloys with *x*_Ti_ ≥ 20 at% were corrected for strengthening by interstitial O.

Min et al.^28^ investigated the strengthening by interstitial O in quenched Ti-9Mo for O concentrations between 0.35 at% and 1.7 at% by using Vickers hardness testing. A simplified Labusch-like strengthening, $H=A\cdot {x}_{{{{{{{{\rm{O}}}}}}}}}^{2/3}+{H}_{{{{{{{{\rm{O}}}}}}}}-{{{{{{{\rm{free}}}}}}}}}$, was fitted to their data. *H*_O−free_ represents the hardness of the O-free alloy. *A* denotes the strengthening parameter which was assumed to be constant within this small O concentration range. The data from Min et al. and the fit are presented in Fig. 4b. The hatched area indicates the range of measured O contents in the present Mo-Ti solid solutions. The resulting strengthening function, with *x*_O_ in at%, is then

3 $$H=\left(1.76+1.23\,{x}_{{{{{{{{\rm{O}}}}}}}}}^{2/3}\right)\,{{\mbox{GPa}}}\,$$

**Fig. 5:**
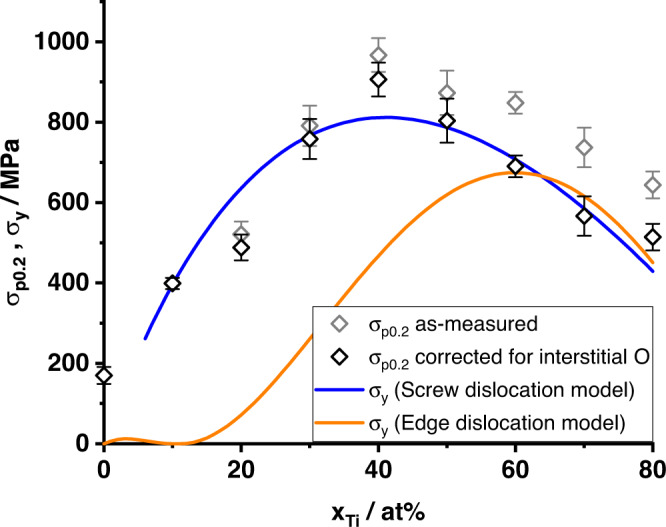
Comparison of offset yield strength to models. 0.2% Offset yield strength *σ*_p0.2_ as measured (gray symbols) and corrected for interstitial O content (black symbols) according to the description in the body text. The solid lines show the predictions for the yield strength *σ*_y_ according to the screw (blue) and edge dislocation model (orange). The screw dislocation model is not applicable in dilute solid solutions.

Based on the good correlation of offset yield strength and Vickers hardness (see section “Correlations of mechanical properties”), the relative increase in hardness as a function of O content was assumed to be equal to the relative increase in offset yield strength, *H*/*H*_O−free_ = *σ*_p0.2_/*σ*_p0.2,O−free_. The *σ*_p0.2_ data was then corrected accordingly based on the O contents determined by HCGE. The obtained offset yield strength and the data corrected for interstitial O are shown in Fig. 5. The corrected data were used for the evaluation of the solid solution strengthening models in what follows.

### Screw dislocation strengthening

Experimentally accessible material properties are not sufficient to apply the screw dislocation model, thus estimates of relevant properties must be made as done in previous applications of the model to other alloys. A concentration-weighted linear mixture of the elemental properties were used for *E*_k_, *E*_v_ and *E*_i_, with the elemental values obtained from experiments or first-principles calculations^24,44–46^. For BCC Ti, the vacancy and self-interstitial formation energies of the hexagonal-closed packed phase^47^ and 2*E*_k_ = 1 eV were used. Most importantly, $\Delta {\tilde{E}}_{{{{{{{{\rm{p}}}}}}}}}$ as a function of composition is required. To achieve a minimal level of fitting for the screw dislocation model, first the collective solute/screw interaction energies in the respective dilute limits are identified as

4 $$\Delta {U}^{{{{{{{{\rm{Ti}}}}}}}}\,{{{{{{{\rm{in}}}}}}}}\,{{{{{{{\rm{Mo}}}}}}}}}={\left(\mathop{\sum}\limits_{i,j}{\left(\Delta {U}_{i,j}^{{{{{{{{\rm{Ti}}}}}}}}\,{{{{{{{\rm{in}}}}}}}}\,{{{{{{{\rm{Mo}}}}}}}}}\right)}^{2}\right)}^{0.5}$$

5 $$\Delta {U}^{{{{{{{{\rm{Mo}}}}}}}}\,{{{{{{{\rm{in}}}}}}}}\,{{{{{{{\rm{Ti}}}}}}}}}={\left(\mathop{\sum}\limits_{i,j}{\left(\Delta {U}_{i,j}^{{{{{{{{\rm{Mo}}}}}}}}\,{{{{{{{\rm{in}}}}}}}}\,{{{{{{{\rm{Ti}}}}}}}}}\right)}^{2}\right)}^{0.5}$$

where $\Delta {U}_{i,j}^{{{{{{{{\rm{Ti}}}}}}}}\,{{{{{{{\rm{in}}}}}}}}\,{{{{{{{\rm{Mo}}}}}}}}}$ describes the interaction energy of a single Ti atom at position *i*, *j* in the core of a pure Mo screw dislocation and vice versa for $\Delta {U}_{i,j}^{{{{{{{{\rm{Mo}}}}}}}}\,{{{{{{{\rm{in}}}}}}}}\,{{{{{{{\rm{Ti}}}}}}}}}$. It is assumed that each collective interaction energy scales linearly with solute concentration,

6 $$\Delta {U}^{{{{{{{{\rm{Ti}}}}}}}}-{{{{{{{\rm{Mo}}}}}}}}}={x}_{{{{{{{{\rm{Mo}}}}}}}}}\,\Delta {U}^{{{{{{{{\rm{Ti}}}}}}}}\,{{{{{{{\rm{in}}}}}}}}\,{{{{{{{\rm{Mo}}}}}}}}}\quad \,{{\mbox{and}}}\,\quad \Delta {U}^{{{{{{{{\rm{Mo}}}}}}}}-{{{{{{{\rm{Ti}}}}}}}}}={x}_{{{{{{{{\rm{Ti}}}}}}}}}\,\Delta {U}^{{{{{{{{\rm{Mo}}}}}}}}\,{{{{{{{\rm{in}}}}}}}}\,{{{{{{{\rm{Ti}}}}}}}}}$$

Following the definition from Eq. (8) and assuming the collective solute/screw interaction energies in both dilute solid solutions are equal, Δ*U*^Ti in Mo^ = Δ*U*^Mo in Ti^, the interaction energy parameter is then

7 $$\Delta {\tilde{E}}_{{{{{{{{\rm{p}}}}}}}}}=\Delta {U}^{{{{{{{{\rm{Ti}}}}}}}}\,{{{{{{{\rm{in}}}}}}}}\,{{{{{{{\rm{Mo}}}}}}}}}\sqrt{(1-{x}_{{{{{{{{\rm{Ti}}}}}}}}}){x}_{{{{{{{{\rm{Ti}}}}}}}}}}$$

Using the stated estimates for the material properties, the quantity Δ*U*^Ti in Mo^ remains as single unknown parameter in the screw dislocation model (Eqs. (9)–(11)). Using a non-linear least-squares algorithm, the model was fitted to the O-corrected *σ*_p0.2_ data between 10 and 80 at% Ti, yielding Δ*U*^Ti in Mo^ = 150 meV. Figure 5 shows the predictions of the fitted screw dislocation model compared to the O-corrected *σ*_p0.2_ data.

The fitted value of Δ*U*^Ti in Mo^ is similar in magnitude to the DFT-computed value Δ*U*^Ta in W^ = 137 meV for refractory, binary Ta-W^18^. The direct calculation of the interaction energy of Ti solutes in Mo by first-principles is possible, but well outside of the scope of this work. The value of $\Delta {\tilde{E}}_{{{{{{{{\rm{p}}}}}}}},50{{{{{{{\rm{Ti}}}}}}}}}=$ 75 meV for Mo-50Ti falls in between reported values for refractory, binary W-50Ta and Nb-50Ti (84 and 55 meV, respectively)^18^. Qualitatively, all these relative values are reflected in the Vickers hardness data published in ref. ^35^.

If a proposed cross-kink annihilation process^48–50^ was included, even though not expected to operate at room temperature, the cross-kink strength contribution in Mo-Ti would be lower than assumed assumed here and the fitted value of Δ*U*^Ti in Mo^ would be larger. According to Eq. (7), the values of the parameter $\Delta {\tilde{E}}_{{{{{{{{\rm{p}}}}}}}}}$ are symmetrical about a maximum value at 50 at% Ti. Thus, the yield strength is also expected to be symmetrical about a maximum at 50% Ti (disregarding small effects from the different Burgers vector lengths). However, the energy required to form self-interstitials decreases strongly from around 7.4 eV in Mo^46^ to 2.3 eV in Ti^47^, which results in a strong decrease of the strengthening contribution by cross-kink breaking with increasing Ti concentration (see Eq. (10)). Thus, the maximum in yield strength is shifted to smaller Ti contents, consistent with the peak observed at around 40 at% in the experimental data.

The model assumes a spontaneously kinked dislocation, and so is not appropriate in the dilute limit (roughly 5% and below) where strength is controlled by double-kink nucleation and kink glide. Thus, the predictions are not shown in this dilute regime. With additional input parameters, e.g., by DFT simulations, the dilute limit can be modeled^51^, but this is not within the scope of this work.

The fitted predictions are in good agreement with the experimental data overall. Thus, the fitted screw dislocation model can reasonably capture the experimental trends across the entire composition range studied here.

### Edge dislocation strengthening

The edge dislocation model depends on the collective misfit parameter *δ*_CV_, that has been directly derived from experiments, and on the experimentally available alloy elastic stiffnesses. As presented in Fig. 2, the concentration-weighted averages of the *C*_*ij*_ of the elements lead to an estimate of the polycrystalline Young’s moduli in good agreement with the data. Accordingly, the shear moduli $\bar{G}$ and Poisson’s ratios $\bar{\nu }$ are calculated using the same concentrated-weighted averages ${\bar{C}}_{ij}$ and the formulae given in Eq. (2). Thus, no inputs to the model are adjusted to match the experimental strength; rather, the model predicts the strength using basic experimentally-derived material properties.

Using the above material parameters, all consistent with experiments, predictions by the edge dislocation model are shown in Fig. 5. The strength is significantly underestimated below 60 at% Ti, which correlates with the very small misfit parameter at low Ti content. Thus, the alloy strength must solely be controlled by screw dislocations in this low-Ti regime. At concentrations of 60 at% Ti and higher, the edge dislocation model shows very good agreement with experiments without any fitting, and agrees similarly well as the *fitted* screw dislocation model. Thus, it is concluded that edge dislocation strengthening is significant in Mo-Ti solid solutions at high Ti contents and can control the alloy strength.

The peak in predicted strengthening of the edge model occurs at ≈60% Ti while the peak in the misfit parameter (see Fig. 1b) is observed at over 70% Ti. The difference arises because the strengthening also depends on the elastic moduli, and the elastic moduli are steadily decreasing with increasing Ti content. The peak strengthening is thus shifted to lower Ti values relative to the peak misfit value.

The strength prediction at 50% Ti can vary notably depending on the numerical fit to the lattice parameter data. Use of a fourth-order polynomial yields a slightly steeper slope at 50% and, consequently, an approximately 10% increase in strength, pushing the prediction closer to experiment (not shown here). A linear fit to the data above 40% Ti leads to an even steeper slope and again higher strengthening. Thus, additional lattice parameter data for concentrated solid solutions is necessary for more accurate predictions in this composition range.

Good agreement between prediction and experiment is achieved for values of *δ*_CV_ ≥ 0.028. This value is slightly lower than the recently-suggested value of 0.035 at which edge dislocation-based strengthening dominates over screw-based one^18^. The latter is a rough guideline that neglects the role of elastic moduli. The high modulus of Mo provides a fairly high modulus for the Mo-Ti alloys even at high Ti content, enabling edge strengthening to achieve parity with screw dislocation strengthening at the lower value of *δ*_CV_.

## Conclusions

This work presents a careful experimental study of Mo-Ti solid solutions to assess the application of solid solution strengthening models and strengthening contributions in concentrated alloys. The results indicate a possible transition between strength controlled by screw and edge dislocation motion. From the data and the analysis, the following conclusions can be drawn:

- In Mo-Ti solid solutions, grain size and grain orientation do not influence the strength significantly.
- Unavoidable synthesis-related interstitial O increases the strength of Mo-Ti solid solutions and therefore is accounted for in modeling solid solution strengthening, while oxides formed at grain boundaries do not impact the strength.
- The lattice parameter as a function of Ti concentration shows a strong non-linear behavior. This leads to small misfit parameters at concentrations below 40 at% Ti, but large misfit parameters at 60–80 at% Ti, with the misfit parameter around 50% Ti being very sensitive to the fitting of the data.
- The screw dislocation-based model for solid solution strengthening by Maresca and Curtin^24,25^ describes the yield strength over the entire concentration range in qualitative and quantitative agreement with the experimental results. It requires a single fitting parameter whose value is comparable to those used to match strengths in other BCC solid solution alloys.
- An observed off-centered peak strength is reproduced by the screw dislocation model and can be rationalized by the strong change in self-interstitial formation energy.
- The edge dislocation-based model for solid solution strengthening by Maresca and Curtin^12^ predicts the yield strength in the Mo-Ti system in agreement with the experimental results for solid solutions with *x*_Ti_ ≥ 60 at% without the need of fitting to the strength data.
- The possible transition from yield strength controlled by screw dislocations at low concentrations to yield strength controlled by edge dislocations correlate with the strongly non-linear behavior of the lattice parameter and, hence, large misfit parameters this alloy system. This is consistent with another recent analysis^18^ when the rather large elastic stiffnesses of Mo are taken into account.
- Possible cross-kink annihilation processes not considered here lead to an overestimation of screw dislocation-controlled strength. This further supports the relevance of edge dislocation-based strengthening at high Ti contents.

## Methods

### Synthesis

High purity Mo (99.95%, EvoChem, Germany) and Ti (99.8%, ChemPur, Germany) were melted using an arc melter AM/0.5 (Edmund Bühler GmbH, Germany) to a button shape in a water-cooled Cu crucible under Ar atmosphere (≥99.998%, Air Liquide, France). The furnace chamber was evacuated and flooded with Ar for at least three times prior to synthesis. In the last evacuation cycle, the chamber was pumped to less than 2 × 10^−4^ mbar and for arc-melting an Ar pressure of 600 mbar was used. Each button was flipped and remelted four more times to ensure homogeneity. A Zr getter was liquefied before each step in order to clean the atmosphere from O.

### Structural and compositional analysis

For XRD, a D2 Phaser (Bruker, Germany) in Bragg-Brentano geometry with Cu K *α* radiation was used. The Nelson-Riley approach was used to determine the lattice parameter of each composition^52^. O and N contents were determined using HCGE on at least three samples per composition using a TC600 device (Leco Instrumente GmbH, Germany). To differentiate dissolved O and O bonded in Ti oxides, APT was performed on Mo-10Ti and Mo-80Ti at the center of grains. Five tips of a specimen of each composition were prepared in a Strata400 dual beam SEM/focused ion beam device (Field Electron and Ion Company, USA) using annular milling with Ga^+^ ions at 30 kV with decreasing inner diameter down to 0.2 μm. Final milling with a closed circular pattern was performed at 5 kV acceleration voltage to minimize the layer affected by Ga^+^ ions on the tip surface. APT analyses were conducted in a LEAP 4000X HR (Cameca SAS, France). The device was operated in laser mode (UV laser with *λ* = 355 nm) at a pulse energy of 100 pJ and a pulse repetition rate of 100 or 125 kHz. The temperature was set to 50 K and the standing high voltage was controlled according to a detection rate of 0.3 or 0.5%. APT data were reconstructed and analyzed by the IVAS 3.8.8 software (Cameca SAS, France). The chemical composition was determined using the peak deconvolution analysis to take the possible overlap of peaks into account, especially the overlap of TiO^2+^ and ${\rm{O}}_{2}^{+}$ at 32 u/e.

### Sample preparation

Specimens for compression testing were machined using electric discharge machining to a size of about 5 × 3 × 3 mm^3^. The punch contact faces of these specimens were ground down to SiC grit P2500 paper. Specimens for XRD, Vickers hardness testing and nanoindentation were ground to SiC grit P4000 paper and polished afterwards using a 5:1 mixture of OP-S (Buehler ITW, Germany) and 30% concentrated H_2_O_2_.

### Mechanical testing

Compression tests were performed at room temperature on a universal testing machine type 1478 (ZwickRoell, Germany) at an initial strain rate of $\dot{\varepsilon }=1{0}^{-4}\,{{{{{{{{\rm{s}}}}}}}}}^{-1}$. For each composition, at least three compression tests were analyzed and the offset yield strengths at 0.2% and 5% engineering strain (*σ*_p0.2_ and *σ*_p5_, respectively) were determined. A Q10A+ device (QATM GmbH, Germany) was used for Vickers hardness testing at room temperature using 98 N load (HV10). At least ten indents per sample were averaged. Vickers hardness and yield strength are considered to be marginally affected by the dendritic microstructure of samples with 40 at% Ti and more. The impact is neglected. Though, for nanoindentation, specimens were homogenized in a tube furnace (Carbolite Gero GmbH, Germany) at 1600 °C for 24 h in Ar flow with a heating and cooling rate of 100 °C h^−1^. Room temperature nanoindentation was performed on a Nanoindenter XP (MTS, USA) with a Berkovich-tip in continuous stiffness measurement mode (CSM) using an amplitude of 2 nm and a frequency of 45 Hz at a load rate $\dot{P}/P=2\dot{\varepsilon }=0.05\,{{{{{{{{\rm{s}}}}}}}}}^{-1}$ up to a depth of ca. 1.5 μm. A distance of at least 50 μm was kept between indents. The same distance was kept to grain boundaries detected in the optical microscope. Hardness and Young’s modulus were determined according to the Oliver-Pharr method (assuming *ν* = 0.3 for all alloys)^53^. No significant indentation size effect was observed over the indentation depth. Therefore, the data were averaged in depths between 500 and 1200 nm. At least 15 indents were analyzed per specimen.

### Solid solution strengthening models

All strengthening models for random alloys share the same conceptual framework. Dislocations in a random alloy become spontaneously wavy in order to minimize their energy. Dislocation segments of a characteristic length *ζ*_c_ are then pinned in the low-energy regions and must be thermally activated to glide past neighboring high-energy regions at another characteristic distance *w*_c_^24,25,54^. The fluctuations in local composition create fluctuations in local dislocation energies due to the solute/dislocation interaction energies *U*^*n*^(*x*_*i*_, *y*_*j*_) between a solute of type *n* at position (*x*_*i*_, *y*_*j*_) and a dislocation at the origin with line direction along *z*. For edge dislocations, the *U*^*n*^(*x*_*i*_, *y*_*j*_) can be estimated using elasticity theory as *U*^*n*^(*x*_*i*_, *y*_*j*_) = − *p*(*x*_*i*_, *y*_*j*_)Δ*V*_*n*_ where *p*(*x*_*i*_, *y*_*j*_) is the pressure field of the dislocation at the position of the solute and Δ*V*_*n*_ is the misfit volume of the solute in the alloy^12^.

For screw dislocations, which generate no long range pressure field, there is no reduction of *U*^*n*^(*x*_*i*_, *y*_*j*_) to a simple form. Historically, a modulus misfit between solute and matrix has been used^16,22^. For both screw and edge dislocations, due to interaction of the dislocation with many solutes around a dislocation segment of length *ζ*_c_, the relevant quantity for strengthening is a collective energy per unit length

8 $$\Delta {\tilde{E}}_{{{{{{{{\rm{p}}}}}}}}}({w}_{{{{{{{{\rm{c}}}}}}}}}) = 	 \, {\left[\mathop{\sum}\limits_{i,j,n}{x}_{n}{\left(\Delta {U}_{ij}^{n}\right)}^{2}\right]}^{1/2}\\ \Delta {U}_{ij}^{n} = 	 \, {U}^{n}({x}_{i}-{w}_{{{{{{{{\rm{c}}}}}}}}},{y}_{j})-{U}^{n}({x}_{i},{y}_{j})$$

where *x*_*n*_, (*n* = 1, …, *N*), are the concentrations of the *N* alloying elements. The characteristic amplitude of the waviness is *w*_c_ = *a*_P_ for screw dislocations, where *a*_P_ is the Peierls’ valley spacing, and emerges naturally for edge dislocations from the model^12^.

For screw dislocations, the wavy structure consists of atomic kinks along the dislocation. The characteristic length is derived to be ${\zeta }_{{{{{{{{\rm{c}}}}}}}}}={\left(1.08\frac{{E}_{{{{{{{{\rm{k}}}}}}}}}}{\Delta {\tilde{E}}_{{{{{{{{\rm{p}}}}}}}}}}\right)}^{2}\,b$ where *E*_k_ is the kink formation energy and *b* the length of the Burgers vector. Dislocation motion corresponds to lateral glide of kinks across the high-energy regions created by the solute fluctuations, and these barriers must be overcome by thermal activation^24,25^. An experimentally-set enthalpy $\Delta H={k}_{{{{{{{{\rm{B}}}}}}}}}\,T\,\,{{\mbox{ln}}}\,\left({\dot{\varepsilon }}_{0}/\dot{\varepsilon }\right)$ is used, where $\dot{\varepsilon }$ denotes the strain rate and *T* the temperature of the experiment. ${\dot{\varepsilon }}_{0}=1{0}^{4}\,{{{{{{{{\rm{s}}}}}}}}}^{-1}$ is a reference strain rate. Then, the stress to move the kinks can be described as)

9 $${\tau }_{{{{{{{{\rm{k}}}}}}}}}\left(\dot{\varepsilon },T\right) = 	 \, {\tau }_{{{{{{{{\rm{b}}}}}}}}}+{\tau }_{{{{{{{{\rm{c}}}}}}}}}\left[3.26{\left(\frac{\Delta H}{\Delta {\tilde{E}}_{{{{{{{{\rm{p}}}}}}}}}}-0.06\frac{{E}_{{{{{{{{\rm{k}}}}}}}}}}{\Delta {\tilde{E}}_{{{{{{{{\rm{p}}}}}}}}}}+1.07\sqrt{{w}_{{{{{{{{\rm{k}}}}}}}}}/b}\right)}^{-1}-1.58\,\frac{\Delta {\tilde{E}}_{{{{{{{{\rm{p}}}}}}}}}}{{E}_{{{{{{{{\rm{k}}}}}}}}}}\right],\quad {\tau }_{{{{{{{{\rm{k}}}}}}}}} \, > \, {\tau }_{{{{{{{{\rm{b}}}}}}}}},\\ {\tau }_{{{{{{{{\rm{k}}}}}}}}}\left(\dot{\varepsilon },T\right) = 	 \, {\tau }_{{{{{{{{\rm{b}}}}}}}}}-{\tau }_{{{{{{{{\rm{c}}}}}}}}}\left[\frac{\Delta {\tilde{E}}_{{{{{{{{\rm{p}}}}}}}}}^{2}}{1.59\,{E}_{{{{{{{{\rm{k}}}}}}}}}^{2}}\left(\frac{\Delta H}{\Delta {\tilde{E}}_{{{{{{{{\rm{p}}}}}}}}}}-2.12\,\frac{{E}_{{{{{{{{\rm{k}}}}}}}}}}{\Delta {\tilde{E}}_{{{{{{{{\rm{p}}}}}}}}}}+1.07\sqrt{{w}_{{{{{{{{\rm{k}}}}}}}}}/b}\right)\right],\quad {\tau }_{{{{{{{{\rm{k}}}}}}}}} \, < \, {\tau }_{{{{{{{{\rm{b}}}}}}}}}.$$

where *w*_k_ is the kink width (typically ~ 10 *b*), ${\tau }_{{{{{{{{\rm{c}}}}}}}}}=\Delta {\tilde{E}}_{{{{{{{{\rm{p}}}}}}}}}/({a}_{{{{{{{{\rm{P}}}}}}}}}\,{b}^{2})$ is a characteristic stress, and ${\tau }_{{{{{{{{\rm{b}}}}}}}}}=1.08\,{E}_{{{{{{{{\rm{k}}}}}}}}}/\left({a}_{{{{{{{{\rm{P}}}}}}}}}\,b\,{\zeta }_{{{{{{{{\rm{c}}}}}}}}}\right)$ is a backstress-like term^24^.

Kinks can form on different {110} slip planes along the same screw dislocation, and the intersections of such kinks on different planes form cross-kinks. For dislocation motion, these cross-kinks need to be overcome. One possible mechanism is the breaking of cross-kinks under simultaneous formation of point defects. At elevated temperatures, some simulations indicate that cross-kinks might also glide and annihilate with cross-kinks of opposite orientation^48–50^. However, there is no theory for this behavior and we restrict our analysis here to room temperature, where this annihilation mechanism is not expected to operate. Cross-kink strengthening can then be calculated as

10 $${\tau }_{{{{{{{{\rm{xk}}}}}}}}}\left(\dot{\varepsilon },T\right)=\frac{\pi {E}_{{{{{{{{\rm{v}}}}}}}}/{{{{{{{\rm{i}}}}}}}}}}{{a}_{{{{{{{{\rm{P}}}}}}}}}\,b\,{\zeta }_{{{{{{{{\rm{v}}}}}}}}/{{{{{{{\rm{i}}}}}}}}}}\left[1-{\left(\frac{\Delta H}{{E}_{{{{{{{{\rm{v}}}}}}}}/{{{{{{{\rm{i}}}}}}}}}}\right)}^{2/3}\right]$$

where *E*_v/i_ and *ζ*_v_ = 7.5 *ζ*_c_ and *ζ*_i_ = 15 *ζ*_c_ are the vacancy and self-interstitial formation energies and their corresponding characteristic lengths, respectively. The larger of the vacancy- and self-interstitial-controlled strength is the decisive mechanism in cross-kink breaking.

The total strength due to screw dislocation motion at room temperature and above is the sum of the above two strength contributions. The yield strength is the shear strength multiplied by the Taylor factor of 2.74 for screw dislocation slip in a random BCC polycrystal by pencil-glide (〈111〉 slip directions, multiple sets of slip planes)^55^, leading to

11 $${\sigma }_{{{{{{{{\rm{y}}}}}}}}}\left(\dot{\varepsilon },T\right)=2.74\,\left({\tau }_{{{{{{{{\rm{xk}}}}}}}}}\left(\dot{\varepsilon },T\right)+{\tau }_{{{{{{{{\rm{k}}}}}}}}}\left(\dot{\varepsilon },T\right)\right)$$

Predictions of *σ*_y_ thus require (i) the alloy Burgers vector length *b*, (ii) the kink formation energy *E*_k_, (iii) the vacancy and the self-interstitial formation energies *E*_v/i_, and (iv) the solute/dislocation interaction energy parameter $\Delta {\tilde{E}}_{{{{{{{{\rm{p}}}}}}}}}$.

The strengthening of edge dislocations was also developed in terms of $\Delta {\tilde{E}}_{{{{{{{{\rm{p}}}}}}}}}$. Maresca et al.^12^ then showed that using the elasticity approximation *U*^*n*^(*x*_*i*_, *y*_*j*_) = − *p*(*x*_*i*_, *y*_*j*_)Δ*V*_*n*_ enabled an analytic solution for the strengthening. They then used the form of that analytic solution to fit numerical prefactors to a number of full simulations on model HEAs, achieving good results between the analytic model and the simulations irrespective of the number of constituents and chemical complexity^12^. The model is thus applicable to the binary Mo-Ti system. In this analytic model, the zero Kelvin strength and the zero Kelvin energy barrier are obtained as

12 $${\tau }_{{{{{{{{\rm{y}}}}}}}}0}=0.040\,{\alpha }^{-1/3}\bar{G}{\left(\frac{1+\bar{\nu }}{1-\bar{\nu }}\right)}^{4/3}{\left[\frac{{\sum }_{n}{x}_{n}\Delta {V}_{n}^{2}}{{b}^{6}}\right]}^{2/3}$$

13 $$\Delta {E}_{{{{{{{{\rm{b}}}}}}}}}=2.00\,{\alpha }^{1/3}\,\bar{G}\,{b}^{3}{\left(\frac{1+\bar{\nu }}{1-\bar{\nu }}\right)}^{2/3}{\left[\frac{{\sum }_{n}{x}_{n}\Delta {V}_{n}^{2}}{{b}^{6}}\right]}^{1/3}$$

where $\bar{G}$ and $\bar{\nu }$ are the isotropic alloy elastic constants and *α* = 1/8 is a line tension parameter where the line tension is $\Gamma =\alpha \,\bar{G}\,{b}^{2}$. Then, edge dislocation-based strengthening is controlled mainly by the collective solute misfit parameter ${\delta }_{{{{{{{{\rm{CV}}}}}}}}}=\frac{1}{3\bar{V}}\sqrt{{\sum }_{n}{x}_{n}\,\Delta {V}_{n}^{2}}$ where *V* is the average atomic volume^17^. The numerical factors 0.040 and 2.00 appearing in the above equations are the values that enabled the analytic theory to match many full simulation results.

Thermal activation theory leads to the finite-temperature, finite strain rate yield strength that can be well-described over a wide temperature range by the ad hoc form^12^

14 $${\sigma }_{{{{{{{{\rm{y}}}}}}}}}\left(T,\dot{\varepsilon }\right)=3.06{\tau }_{{{{{{{{\rm{y}}}}}}}}0}\exp \left[-\frac{1}{0.55}{\left(\frac{{k}_{{{{{{{{\rm{B}}}}}}}}}T\ln \left(\frac{\dot{{\varepsilon }_{0}}}{\dot{\varepsilon }}\right)}{\Delta {E}_{{{{{{{{\rm{b}}}}}}}}}}\right)}^{0.91}\right]$$

for a random-textured polycrystalline sample, where 3.06 is the Taylor factor for edge dislocation slip in BCC alloys^55^ (〈111〉 slip directions, $\{1\overline{1}0\}$ slip planes). Thus, only lattice parameter data and elastic moduli are required as material specific inputs. No fitting of the model is required to match the strength data in any specific alloy.

### Microstructure and composition

The microstructure was imaged on a Zeiss EVO50 scanning electron microscope (Carl Zeiss AG, Germany) using a backscattered electron (BSE) detector at 25 kV acceleration voltage. The line intercept method was applied to these images to determine the average grain size.

**Fig. 6:**
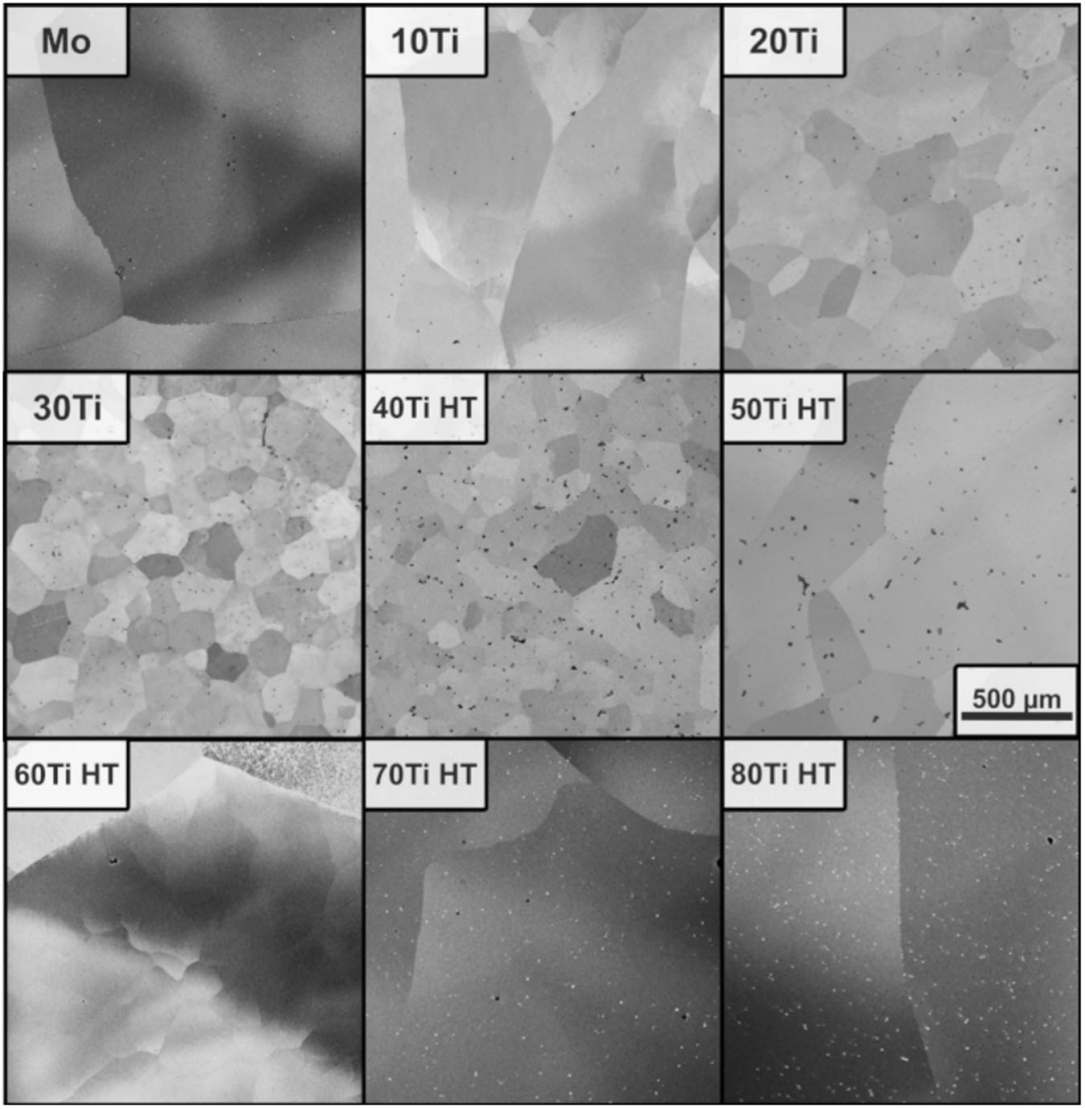
Microstructure. SEM-BSE micrographs of the microstructures for Mo-Ti solid solutions. Alloys containing 40 at% Ti and more are shown after the homogenization treatment (HT). All micrographs are presented at the same magnification.

In these micrographs, samples with Ti concentrations below 40 at% showed a homogeneous element distribution, while a dendritic microstructure was detected in alloys containing 40 at% Ti and more. After the homogenization treatment, these samples also showed a homogeneous microstructure, see Fig. 6. Grain sizes were at least 200 μm in the as-cast state for all alloys, which increased during homogenization.

The composition of each sample was confirmed using the average of at least five energy-dispersive X-ray (EDX) investigations at an acceleration voltage of 10 kV using a Si drift detector (Thermofisher Scientific Inc., USA) mounted on a Zeiss EVO50 scanning electron microscope (Carl Zeiss AG, Germany).

The EDX analyses of the samples show good agreement between the desired and the measured composition. The largest deviation was 1.5 at% in the sample Mo-40Ti, see Table 2. In APT, the Mo-10Ti sample revealed an average Ti content of 10.1 at% after peak deconvolution, which confirms the EDX results for this sample. In the APT measurements of Mo-10Ti, no significant amount of O is detected. In Mo-80Ti, the Ti content is lower than the content determined by EDX, but the O content is identical for both methods within the given errors.

**Table 2:** Chemical composition of the Mo-Ti alloys using EDX, APT and HCGE.

Desired	*x*_Ti_/at%		*x*_O_/at-ppm		*x*_N_/at-ppm	
*x*_Ti_/at%	EDX	APT	HCGE	APT	HCGE	APT
10	9.4 ± 0.5	10.1	<496	0^a^	104^a^ ± 0	0^a^
20	19.6 ± 0.5	–	955 ± 577	–	123 ± 44	–
30	29.0 ± 0.5	–	617 ± 113	–	111 ± 41	–
40	38.5 ± 0.5	–	959 ± 187	–	137 ± 59	–
50	50.0 ± 0.5	–	1232 ± 372	–	113 ± 10	–
60	60.3 ± 0.5	–	3268 ± 690	–	110 ± 36	–
70	70.0 ± 0.5	–	4316 ± 924	–	133 ± 42	–
80	80.0 ± 0.5	76.5	3626 ± 787	3300	185 ± 58	<100

Balance is Mo. Values marked with ^a^ were below detection limit. Errors in APT measurements are below the given decimal places and therefore omitted. For all other methods, a conservative estimate of error is given.

A frequency distribution analysis (bin size 100) was performed on Mo-10Ti and Mo-80Ti to determine the Pearson correlation coefficient (PCC) of element distribution. A value of 0 indicates a random distribution of elements, while a value of 1 indicates a fully ordered arrangement^56^. The results are all close to 0, which indicates a random distribution, see Table 1. Still, the Pearson coefficient is higher in Mo-80Ti, indicating a larger amount of order. A nearest-neighbor analysis (NNA) was performed to determine the average distance $\bar{d}$ to nearest neighbors of the same ion species compared to a random data set. The distance between same-species neighbors ${\bar{d}}_{{{{{{{{\rm{ss}}}}}}}}}$ was slightly smaller than for randomized data ${\bar{d}}_{{{{{{{{\rm{rs}}}}}}}}}$, see Table 1. However, the difference between the data sets are too small to perform a cluster analysis with meaningful results. Note that both Mo and Mo_2_ ions were detected and analyzed separately by the IVAS software. In the table, only results for the dominant Mo are presented.

### Correlations of mechanical properties

**Fig. 7:**
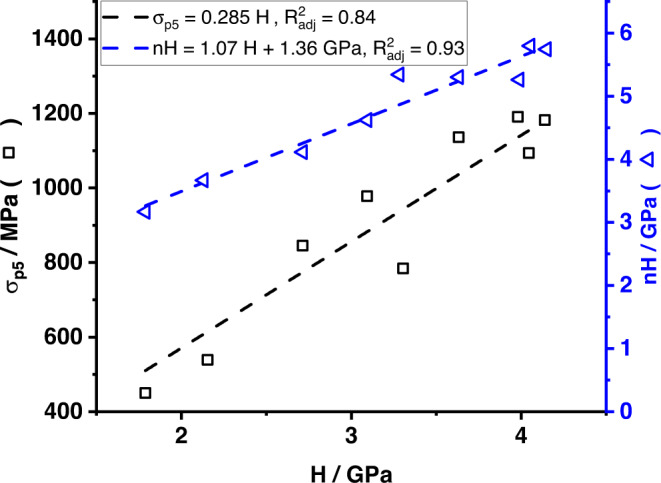
Correlations between mechanical properties. Correlation graphs between the Vickers hardness *H* (horizontal axis) and *σ*_p5_ (black squares, left axis) and *H* and nanohardness *nH* (blue triangles, right axis).

Nanohardness *nH*, Vickers hardness measurements *H* and 5% offset yield strength *σ*_p5_ show similar trends, see Fig. 3. In order to quantify the similarities, the correlations between *nH* and *H* and *σ*_p5_ and *H* were determined, see Fig. 7. Both graphs yield a very good correlation, ${R}_{{{{{{{{\rm{adj}}}}}}}}}^{2}=0.93$ and ${R}_{{{{{{{{\rm{adj}}}}}}}}}^{2}=0.84$, respectively. From the correlation graph between *σ*_p5_ and *H*, the factor 0.285 is extracted, which is close to the suggested value of 0.3^42^. The offset of 1.36 GPa between *nH* and *H* is attributed to the indentation size effect^40^.

## Data Availability

The data presented in this study are available in KITopen at 10.5445/IR/1000157205 under CC BY-SA 4.0 license. The Matlab code is stored at KITopen 10.5445/IR/1000157208 under CC BY-SA 4.0 license.
